# Death from Chloroform in a Dentist’s Chair

**Published:** 1889-06

**Authors:** 


### Selections.

### Death from Chloroform in a Dentist's Chair.

According to press dispatches a young lady died in a dentist's chair in Norwalk, Ohio, on February 21, after having had chloroform administered for the extraction of a tooth. It is stated that she " partly recovered from the drug," when " she started to rise from the chair, but suddenly sank back and died." From the meagre information conveyed in the press dispatches one can not know whether the chloroform was administered by the dentist himself, or whether the administrator was a physician, or a person sufficiently skilled to administer chloroform ; but one can scarcely conceive of a skillful anæsthetizer giving chloroform to any one in a dentist's chair under any circumstances. A person that would give chloroform to a patient in a chair, would probably not take the other necessary precautions when about to give chloroform, such as removing all sources of constriction of the body.

Is it not time that dentists, and physicians also, learn that chloroform is a very dangerous drug when given improperly ? With the other means of anæsthesia that we have, is the use of chloroform for tooth-extraction justifiable ? The fact that teeth have been extracted under chloroform, and without injury to the patient, does not justify its use for this purpose. The surgeon that would seat a patient fully clothed in a chair, and give chloroform to amputate a finger, or open an abscess, would be guilty of negligence little, if any, short of criminality. We are too much inclined to excuse such blunders, hoping that the blunderers have been taught a salutary lesson, which, however, is of no benefit to the person that has come to an untimely death. There should be more care, in dealing with the ills to which the flesh is heir, that the patient survive the treatment.

